# Changes in Urinary Biomarkers of Organ Damage, Inflammation, Oxidative Stress, and Bone Turnover Following a 3000-m Time Trial

**DOI:** 10.3390/antiox10010079

**Published:** 2021-01-09

**Authors:** Takaki Tominaga, Sihui Ma, Kaoru Sugama, Kazue Kanda, Chiaki Omae, Wonjun Choi, Shunsuke Hashimoto, Katsuhiko Aoyama, Yasunobu Yoshikai, Katsuhiko Suzuki

**Affiliations:** 1 Graduate School of Sport Sciences, Waseda University, Tokorozawa 359-1192, Japan; chappy-ld312@moegi.waseda.jp (C.O.); wonwon11110@fuji.waseda.jp (W.C.); 2 Research Fellow of Japan Society for the Promotion of Sciences, Tokyo 102-0083, Japan; masihui@toki.waseda.jp; 3 Faculty of Sport Sciences, Waseda University, Tokorozawa 359-1192, Japan; 4 Future Innovation Institute, Waseda University, Shinjuku 162-0041, Japan; k.sugama@kurenai.waseda.jp (K.S.); rurishijimikanda@gmail.com (K.K.); 5 Ortho Corporation, Shibuya 150-0002, Japan; hashimoto@kenko.co.jp (S.H.); aoyama@kenko.co.jp (K.A.); 6 Division of Host Defense, Medical Institute of Bioregulation, Kyushu University, Fukuoka 812-8582, Japan; yoshikai@bioreg.kyushu-u.ac.jp

**Keywords:** urinary biomarkers, cytokines, chemokines, oxidative stress, bone resorption markers, liver-fatty acid binding protein (L-FABP), intestine-fatty acid binding protein (I-FABP), titin N-terminal fragments, acute exercise

## Abstract

Strenuous exercise induces organ damage, inflammation, and oxidative stress. Currently, to monitor or investigate physiological conditions, blood biomarkers are frequently used. However, blood sampling is perceived to be an invasive method and may induce stress. Therefore, it is necessary to establish a non-invasive assessment method that reflects physiological conditions. In the present study, we aimed to search for useful biomarkers of organ damage, inflammation, oxidative stress, and bone turnover in urine following exercise. Ten male runners participated in this study and performed a 3000-m time trial. We measured biomarkers in urine collected before and immediately after exercise. Renal damage markers such as urea protein, albumin, N-acetyl-β-D-glucosaminidase (NAG), and liver-fatty acid binding protein (L-FABP), and an intestinal damage marker, intestine-fatty acid binding protein (I-FABP), increased following exercise (*p* < 0.05). However, a muscle damage marker, titin N-terminal fragments, did not change (*p* > 0.05). Inflammation-related factors (IRFs), such as interleukin (IL)-1β, IL-1 receptor antagonist (IL-1ra), IL-6, complement (C) 5a, myeloperoxidase (MPO), calprotectin, monocyte chemoattractant protein (MCP)-1, and macrophage colony-stimulating factor (M-CSF), increased whereas IRFs such as IL-4 and IL-10 decreased following exercise (*p* < 0.05). IRFs such as tumor necrosis factor (TNF)-α, IL-2, IL-8, IL-12p40, and interferon (IFN)-γ did not change (*p* > 0.05). Oxidative stress markers, such as thiobarbituric acid reactive substances (TBARS) and nitrotyrosine, did not change following exercise (*p* > 0.05) whereas 8-hydroxy-2′-deoxyguanosine (8-OHdG) decreased (*p* < 0.05). Bone resorption markers, such as cross-linked N-telopeptide of type I collagen (NTX) and deoxypyridinoline (DPD), did not change following exercise (*p* > 0.05). These results suggest that organ damage markers and IRFs in urine have the potential to act as non-invasive indicators to evaluate the effects of exercise on organ functions.

## 1. Introduction

Strenuous endurance exercise induces organ damage such as muscle, renal, and intestinal damage [1,2,3,4,5,6,7]. Strenuous endurance exercise also induces systemic inflammation and oxidative stress [1,2,3,4,5,6,7,8,9]. It is well reported that strenuous exercise increases muscle damage markers, such as creatine kinase (CK) and myoglobin, renal functional markers, such as creatinine and blood urea nitrogen (BUN), and intestinal damage markers, such as intestine-fatty acid binding protein (I-FABP) and ileal-bile acid binding protein (I-BABP) in the circulation [1,2,3,4,5,7,8,9]. Exercise also increases inflammation-related factors (IRFs) including pro-inflammatory and anti-inflammatory cytokines, chemokines, and associated inflammatory mediators [1,2,3,4,5,6,7,10,11] together with oxidative stress markers such as thiobarbituric acid reactive substances (TBARS) and nitrotyrosine in the circulation [12,13]. In addition to appearing in the circulation, IRFs and oxidative stress markers are excreted into urine following exercise [1,2,3,4,10,11,14,15,16,17,18]. 

Currently, to monitor or investigate physiological conditions, blood biomarkers have been frequently used. However, blood sampling is perceived to be an invasive method and may induce stress. Adequate blood volume (e.g., venous blood sampling) is not easy in exercise contexts and only a qualified person can collect samples (e.g., medical doctors). Compared to collecting blood, collecting urine samples is easier and non-invasive. Recent studies have reported that following exercise, organ damage markers and IRFs in urine changed alongside these biomarkers in blood [1,2,3,4,10,11,14,15,16,17]. Therefore, to monitor exercise-induced organ damage, inflammation, and oxidative stress, urinary biomarkers have the potential as an alternative to traditional blood-based analysis. In recent years, non-invasive biomarkers from urine in the context of exercise has attracted attention. For example, exploring urinary biomarkers via the urinary proteome has been conducted before and after exercise [19,20,21]. A constant within previous literature is that titin N-terminal fragments have been well-detected in urine samples following eccentric exercise and identified as a reliable urinary biomarker that reflects the degree of muscle damage [21,22,23].

Stress fractures are common in long-distance runners. The decrease of bone mineral density (BMD) is a major risk factor of stress fractures [24]. The change of BMD is caused by an imbalance of bone turnover (the balance between bone formation and bone resorption) whereas, over the long-term, it is necessary to detect the change of BMD. However, the balance of bone turnover can be detected in real-time using bone turnover markers in blood and urine. Although exercise training changes bone turnover, acute exercise also changes bone turnover [25]. Therefore, it is important to monitor bone turnover following exercise and attempt to minimize the risk of stress fractures.

As we described above, the measurements of organ damage, inflammation, oxidative stress, and bone turnover from urinary biomarkers are important in the exercise context and can decrease the psychological and/or physiological stress on subjects. However, the studies investigating the effects of exercise on urinary biomarkers need to be expanded into other exercise contexts. Therefore, this study aimed to identify useful acute biomarkers in urine in relation to organ damage, inflammation, oxidative stress, and bone turnover in response to a short strenuous exercise bout.

## 2. Methods

### 2.1. Subjects

Data for 10 male runners (age: 19.4 ± 1.5 (mean ± SD), height: 172.8 ± 6.6 cm, body mass: 61.3 ± 5.9 kg) are included in this study. The participants were recreational level runners running 10–100 km per month. Tumor necrosis factor (TNF)-α, interleukin (IL)-1β, IL-1 receptor antagonist (IL-1ra), IL-2, IL-4, IL-6, IL-8, IL-10, IL-12p40, interferon (IFN)-γ, complement (C) 5a, myeloperoxidase (MPO), calprotectin, monocyte chemoattractant protein (MCP)-1, macrophage colony-stimulating factor (M-CSF), fractalkine, N-acetyl-β-D-glucosaminidase (NAG), osmotic pressure, specific gravity, creatinine, I-FABP, and titin N-terminal fragments of 7 subjects were obtained from the pre-intervention timepoint in a previously published study where subjects consumed no immune protein and placebo protein [26]. A further three runners’ data were obtained separately. Further analysis of urea nitrogen, uric acid, urine protein, albumin, glucose, pH, cross-linked N-telopeptide of type I collagen (NTX), deoxypyridinoline (DPD), chloride, phosphate, sodium, potassium, calcium, L-FABP, I-BABP, 8-hydroxy-2’-deoxyguanosine (8-OHdG), TBARS, and nitrotyrosine was conducted on all 10 runners.

Written informed consent was obtained from all the participants prior to their enrollment in the study. Inclusion criteria were as follows: (1) participants had to be recreationally trained male runners from Waseda University between the ages of 18–30 years old, (2) participants had to be healthy and free of any known disease determined by a medical history questionnaire, and (3) participants had to abstain from supplemental protein or amino acids for three months prior to participating, but not meal intervention. The experimental protocol was approved by the Ethics Committee of Waseda University (2017-319, date of approval: 11 April 2018), and was conducted under the Declaration of Helsinki.

### 2.2. 3000-m Time Trial

The subjects carried out a 3000-m time trial at an athletic field in the evening (1600–1800 h) after a self-selected warm-up. Meal and fluid intake were not restricted on the days of the experimental trials. The median running time was 597 s (min 562 s, max 717 s). The ratings of perceived exertion (RPE) obtained by using the 6–20 Borg Scale after the warm-up was 10.4 ± 1.8 (mean ± SD) and post-exercise was 18.2 ± 1.0.

### 2.3. Urine Sampling

The detailed protocol of urine sampling has been described previously [26]. The pre-exercise urine samples (Pre) were collected 30–60 min before the 3000-m time trial. The post-exercise urine samples (Post) were collected within 30 min after the run. Fluid intake was prohibited between pre-sampling and post-sampling. The urinary excretion was not restricted before pre-sampling. The collected urine samples were centrifuged at 1000× *g* for 10 min to remove sediments, and the supernatants were stored at −80 °C until the analysis.

### 2.4. Assays for Urinary Biomarkers

Creatinine, osmotic pressure, specific gravity, NAG, albumin, protein, glucose, urea nitrogen, uric acid, chloride, phosphate, sodium, potassium, and calcium were measured by Koutou-Biken Co. (Koutou-Biken Co, Tsukuba, Japan). IL-1β, IL-6, and TNF-α concentrations were measured by a Quantikine high sensitivity (HS) enzyme-linked immunosorbent assay (ELISA) Kit (R&D Systems Inc., Minneapolis, MN, USA). IL-1ra, MCP-1, and M-CSF concentrations were measured by Quantikine ELISA Kit (R&D Systems Inc., Minneapolis, MN, USA). I-FABP and fractalkine concentrations were measured by Duoset ELISA Kit (R&D Systems Inc., Minneapolis, MN, USA). IL-2, IL-4, IL-8, IL-10, IL-12p40, C5a, and IFN-γ concentrations were measured by OptEIA ELISA Kit (Beckton Dickinson Biosciences, San Diego, CA, USA). Calprotectin and MPO concentrations were measured by ELISA Kit (Hycult biotechnology, Uden, The Netherlands). I-BABP concentration was measured by Human FABP6 (Gastrotropin) ELISA Kit (XpressBio, Frederic, MD, USA). L-FABP concentration was measured by High Sensitivity Human L-FABP ELISA Kit (CMIC Company, Tokyo, Japan). NTX and DPD concentrations were measured by the ELISA kit (CUSABIO, Wuhan, China). The concentration of titin N-terminal fragments was measured by ELISA as described previously [21]. The absorbance of the above parameters was measured spectrophotometrically on a VersaMax Microplate Reader (Molecular Devices Inc., San Jose, CA, USA). 8-OHdG and nitrotyrosine concentrations were measured using the ELISA Kit (StressMarq Biosciences, Victoria, BC, USA). The absorbance of 8-OHdG and nitrotyrosine was measured spectrophotometrically on Spectra Max iD5 (Molecular Devices Inc., San Jose, CA, USA). TBARS concentration was measured fluorescently using TBARS Assay Kit (Cayman Chemical Co., Ann Arbor, MI, USA). The fluorescence was measured on FLUOstar Optima plate leader (BMG labtech ltd, Ortenberg, Germany). The concentrations of each parameter were calculated by a comparison with the calculation curve established in the same measurement. To correct urine condensation, the data of each parameter were corrected using urine creatinine. According to the instruction manuals, the intra-assay variation for all ELISA measurements and TBARS was <10%. In the present study, we measured all samples as singlicates because we could not obtain enough sample volume from a few participants to measure a large number of parameters in duplicate. Therefore, we prioritized the acquisition of the number of measurement parameters necessary for hypothesis testing.

### 2.5. Data Analysis

The sample size and effect size (dz) were calculated using the program G*power (available from https://www.psychologie.hhu.de/arbeitsgruppen/allgemeine-psychologie-und-arbeitspsychologie/gpower.html) [27]. 10 subjects were required to detect an effect size of dz = 1.02 with a power of 0.8 and a significance level of 0.05. Data are presented as box-and-whisker plot. Statistical validation was made using a non-parametric Wilcoxon signed-rank test. Statistical significance was defined as *p* < 0.05. Statistical analysis was performed using SPSS version 25.0 (IBM Inc., Armonk, NY, USA).

## 3. Results

### 3.1. Urinary Organ Damage Markers

**Figure 1:**
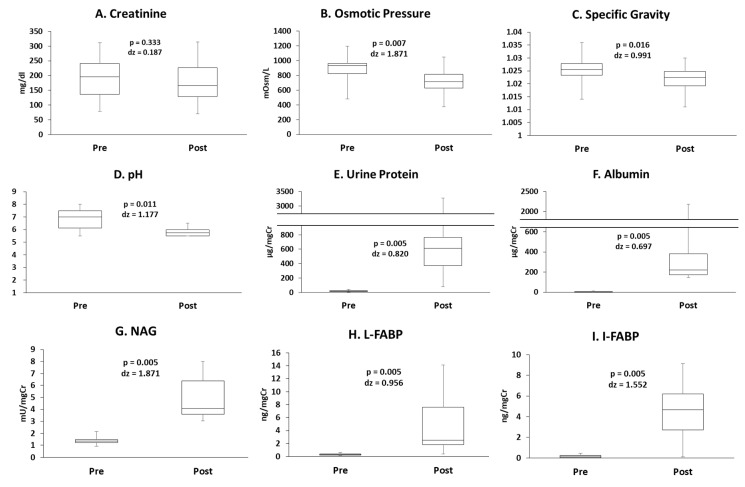

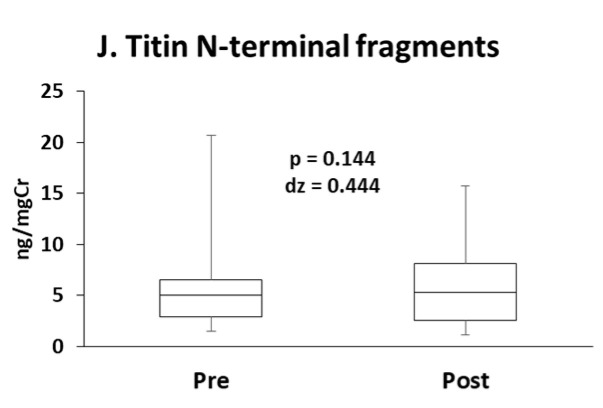
The concentrations of (**A**) creatinine, (**B**) osmotic pressure, (**C**) specific gravity, (**D**) pH, (**E**) urine protein, (**F**) albumin, (**G**) N-acetyl-β-D-glucosaminidase (NAG), (**H**) liver-fatty acid binding protein (I-FABP), (**I**) intestine-fatty acid binding protein (I-FABP), and (**J**) titin N-terminal fragments.

The concentration of urinary creatinine did not change significantly (Figure 1A). Urinary osmotic pressure, specific gravity, and pH decreased following exercise (Figure 1B–D). The concentrations of urine protein, albumin, NAG, L-FABP, and I-FABP increased following exercise (Figure 1E–I). The concentration of titin N-terminal fragments did not change (Figure 1J), whilst I-BABP concentration was below the detection limit of the assay. Uncorrected data and data corrected for urine osmolality as a urine condensation indicator are presented in Supplemental Table S1.

### 3.2. Urinary Inflammation-Related Factors (IRFs)

**Figure 2:**
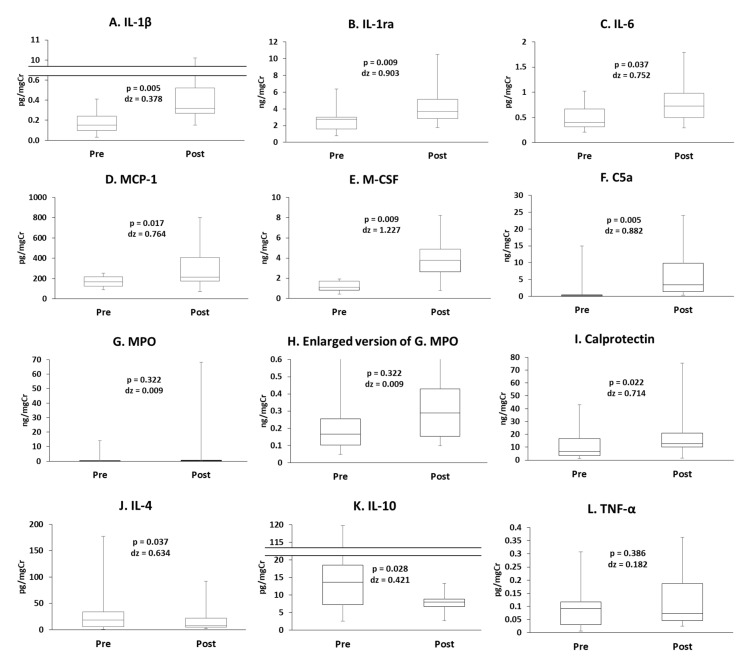

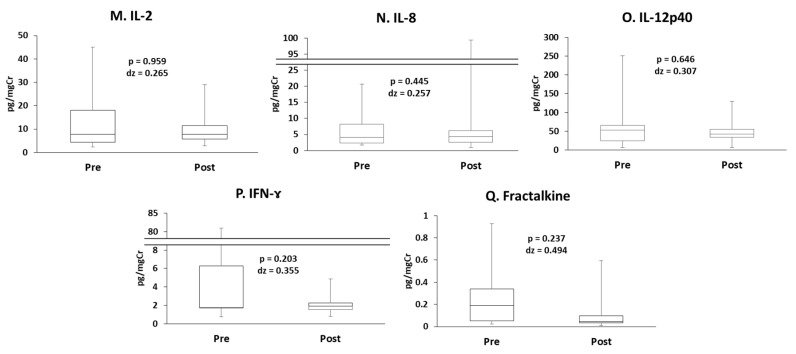
The concentrations of (**A**) interleukin (IL)-1β, (**B**) IL-1 receptor antagonist (IL-1ra), (**C**) IL-6, (**D**) monocyte chemoattractant protein (MCP)-1, (**E**) macrophage colony-stimulating factor (M-CSF), (**F**) complement (C) 5a, (**G**) myeloperoxidase (MPO), (**H**) enlarged version of (**G**), (**I**) calprotectin, (**J**) IL-4, (**K**) IL-10, (**L**) tumor necrosis factor (TNF)-α, (**M**) IL-2, (**N**) IL-8, (**O**) IL-12p40, (**P**) interferon (IFN)-γ, and (**Q**) fractalkine.

The concentrations of IL-1β, IL-1ra, IL-6, MCP-1, M-CSF, C5a, MPO, and calprotectin increased following exercise (Figure 2A–I). However, the concentrations of IL-4 and IL-10 decreased following exercise (Figure 2J,K). The concentrations of TNF-α, IL-2, IL-8, IL12p40, IFN-γ, and fractalkine did not change (Figure 2L–Q). Uncorrected data and data corrected for urine osmolality are presented in Supplemental Table S2.

### 3.3. Oxidative Stress Markers

**Figure 3:**
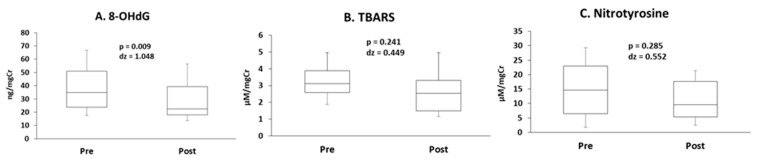
The concentrations of (**A**) 8-hydroxy-2’-deoxyguanosine (8-OHdG), (**B**) thiobarbituric acid reactive substances (TBARS), and (**C**) nitrotyrosine.

The concentration of 8-OHdG decreased following exercise (Figure 3A). The concentrations of TBARS and nitrotyrosine did not change (Figure 3B,C). Uncorrected data and data corrected for urine osmolality are presented in Supplemental Table S3.

### 3.4. Urinary Metabolites

**Figure 4:**
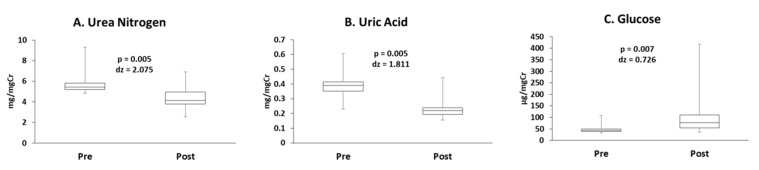
The concentrations of (**A**) urea nitrogen, (**B**) uric acid, and (**C**) urine glucose.

The concentrations of urea nitrogen and uric acid decreased following exercise (Figure 4A,B). However, the concentration of urine glucose increased following exercise (Figure 4C). Uncorrected data and data corrected for urine osmolality are presented in Supplemental Table S4.

### 3.5. Urinary Bone Resorption Markers and Minerals

**Figure 5:**
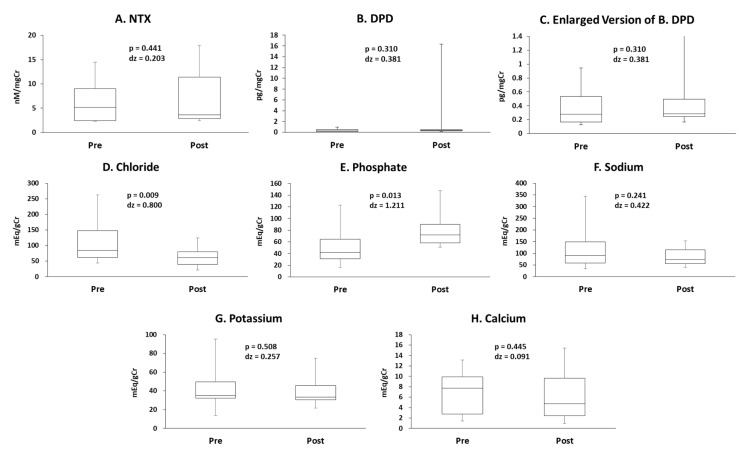
The concentrations of (**A**) cross-linked N-telopeptide of type I collagen (NTX), (**B**) deoxypyridinoline (DPD), (**C**) enlarged version of (**B**), (**D**) chloride, (**E**) phosphate, (**F**) sodium, (**G**) potassium, and (**H**) calcium.

The concentrations of NTX and DPD, which are bone resorption markers, did not change (Figure 5A–C). The concentration of chloride decreased following exercise (Figure 5D). The concentration of phosphate increased following exercise (Figure 5E). The concentrations of sodium, potassium, and calcium did not change (Figure 5F–H). Uncorrected data and data corrected for urine osmolality are presented in Supplemental Table S5.

## 4. Discussion

The main novel finding of this study is that various organ damage markers, IRFs, and metabolites dramatically changed in urine following a 3000-m time trial. In the present study, in addition to traditional exercise-induced organ damage biomarkers such as urine protein, albumin, and NAG, we measured novel organ damage markers such as L-FABP, I-FABP, I-BABP, and titin N-terminal fragments. 

### 4.1. The Changes of Urinary Biomarkers Following Acute Exercise

In the present study, urinary L-FABP levels increased following exercise. Several studies have previously reported changes in urinary L-FABP following exercise [28,29,30]. Hiraki et al. have reported that 20 min of walking did not increase urinary L-FABP levels, which coincides with the absence of increases in urinary albumin and NAG in their reports [28]. However, Kosaki et al. have reported that short incremental maximal exercise increases urinary L-FABP levels, which coincides with the increase of urinary albumin [30]. Kanda et al. have also reported that eccentric exercise does not increase urinary L-FABP levels [29]. These reports and our findings suggest that the increase of urinary L-FABP is dependent on exercise intensity but not muscle damage, and that the response of urinary L-FABP to exercise coincides with urinary NAG and albumin. Sugama et al. have reported that duathlon races increase serum creatinine and urinary albumin, and decrease creatinine clearance and urine volume, which suggest a decrease in renal function. However, the urinary NAG levels in their study were decreased [2]. This report suggests that urinary NAG may not reflect severe proximal tubule cell damage. Therefore, the measurement of urinary L-FABP may be more useful after strenuous, long-duration exercise bouts rather than short-duration exercise bouts. In the present study, urinary I-FABP levels increased following exercise. To our knowledge, this study is the first study to investigate the effect of endurance exercise on urinary I-FABP [26]. However, Kanda et al. have already reported that eccentric exercise does not change urinary I-FABP [29]. Several previous studies have reported that endurance exercise increases I-FABP and I-BABP in the circulation [7,31]. In the present study, urinary I-FABP increased dramatically at about 30-fold following exercise, which may reflect I-FABP excretion in the circulation. In the present study, the concentration of I-FABP in urine following exercise (about 7.2 ng/mL) is even higher than that of the patients with Crohn’s disease (about 0.9 ng/mL) [32], and similar to that of patients diagnosed with acute mesenteric ischemia (about 7 ng/mL) [33]. 

In the present study, no change in titin N-terminal fragments were observed, which suggests a 3000-m time trial did not induce muscle damage, at least immediately after exercise. Titin is one of the proteins within skeletal muscle whose N-terminal fragments have been reported to change in the urine following eccentric exercise similarly to CK or myoglobin [21,22,23]. Therefore, titin N-terminal fragments may be useful as a non-invasive marker that reflects muscle damage. However, no study has investigated the validity of titin N-terminal fragments on endurance exercise. Such investigations are required.

In the present study, various urinary IRFs increased following exercise. In contrast to the present study, Suzuki et al. have reported that no significant change in urinary IRFs was observed immediately after a maximal running exercise test lasting about 10 min [10]. However, urinary IL-1ra, IL-6, M-CSF, and MCP-1 increased one hour after exercise [10]. In contrast to this previous study, changes in various urinary IRFs were observed immediately after exercise in the present study. This difference may be attributed to the higher exercise intensity and a longer sampling time point after exercise (30 min), which provides more time for the biomarkers to be released to the external circulation. 

In the present study, urinary 8-OHdG, TBARS, and nitrotyrosine did not increase immediately after exercise, which suggests that at least immediately after exercise, the 3000-m time trial did not induce systemic oxidative stress. However, urinary IRFs increased dramatically in the present study. Steinberg et al. have reported that the increase of plasma IL-6 and TNF-α levels preceded the increase of plasma TBARS levels [12]. Considering that report and this study together, exercise-induced systemic oxidative stress does not seem to precede the inflammatory response. In the present study, urinary 8-OHdG decreased following exercise. One of the reasons of this decrease may attribute to exercise-induced renal dysfunction. In the present study, the 3000-m time trial did decrease urine specific gravity, which suggests a decrease in urine concentrating ability. Although not significant, TBARS and nitrotyrosine did show a tendency to decrease. Therefore, urinary oxidative stress markers may have decreased following exercise due to a reduced urine concentrating ability.

In the present study, uric acid and urea nitrogen decreased following exercise. Urea nitrogen is a metabolite of protein, and uric acid is a metabolite of purines, which are excreted into urine normally at rest. Acute exercise increases BUN in circulation [1,4] and this increase is attributed to renal dysfunction such as a decreased glomerular filtration rate, which is accompanied by a decrease in urea nitrogen excretion [34]. Therefore, the reduction of urea nitrogen excretion reflects renal dysfunction. Similarly, uric acid may be another pivotal metabolite that reflects renal dysfunction because the reduction of uric acid clearance following acute exercise coincides with the reduction of creatinine clearance and total urine output [35].

The bone resorption markers, NTX and DPD, did not change following exercise here. This result is consistent with other previous studies that circulating NTX was unchanged immediately after high-intensity interval exercise [36,37] and urinary DPD did not change immediately after resistance exercise [38,39]. In the present study, calcium excretion did not change following exercise whereas phosphate excretion increased, which are both constituents of the bone. Therefore, it is assumed that calcium homeostasis was not perturbated and calcium liberation from bone was not induced. Considering the increase of phosphate excretion following exercise, one of the sources of phosphate may be the bone. However, NTX, DPD, and calcium did not change following exercise. Therefore, the increase of phosphate may be independent of bone resorption. Many studies have reported that endurance exercise increases circulating phosphate concentration [40,41] and the source of phosphate is contracting muscles [42]. Therefore, muscle-derived phosphate may contribute to the increase of urinary phosphate in the present study.

One possible mechanism of exercise-induced organ damage is decreased blood flow to the organ. The gastrointestinal hypoperfusion (ischemia) induces intestinal damage and increases circulatory I-FABP [7,31]. Similarly, renal damage is also induced by a decrease in renal blood flow [7,35]. In the present study, the 3000-m time trial conducted is considered to be high-intensity exercise. Therefore, the blood flow of internal organs may have decreased, inducing internal organ damage. In contrast to organ damage, the mechanisms of exercise-induced inflammatory response are unclear [6]. Damaged organ-derived IRFs and gut-derived endotoxins are candidates for triggering exercise-induced inflammation [6,10,31].

### 4.2. The Problems of Urinary Biomarkers as a Non-Invasive Method in the Situation of Exercise

While urinary sampling for analysis is easy and non-invasive, one problem is that urinary IRF levels are influenced by renal damage. In previous studies, changes in circulating IRFs have displayed similar behaviors to those in urine following exercise [1,2,3,4,10,43], which suggests that changes in IRFs in the urine may reflect those in the circulation. However, Sugama et al. have reported that, in subjects experiencing strong exercise-induced renal damage, the changes of MCP-1 in urine differed from that in the circulation [2]. This suggests that damaged kidney-derived MCP-1 contributes to the increase of urinary MCP-1 levels. Furthermore, it is also reported that an increase in urinary calprotectin was observed in intrinsic acute kidney injury, but not observed in prerenal acute kidney injury [44]. Therefore, it is necessary to investigate which IRFs are influenced by exercise-induced renal damage.

Urine condensation affected by dehydration or fluid intake is an important problem to evaluate biomarkers in urine. However, the best methods to correct urine condensation from spot urine collections as completed here are not established in the exercise context. This is an important consideration since considering corrected urinary IRFs alongside uncorrected urinary concentrations, urinary concentration with creatinine correction, the gross amount (uncorrected concentration × urine volume), and the gross amount per minute (urinary excretion rate), has been reported to lead to obtaining different conclusions [2]. Since creatinine is a metabolite of creatine phosphate and creatine contributes to regulate ATP generation [45], the behavior of creatinine excretion following exercise may be different from the behavior at rest. Therefore, creatinine correction may not be the best method to correct spot urine in the situation of exercise. Several studies have tried correction of spot urine using not only urinary creatinine but also urinary osmotic pressure and urinary cystatin C. However, the best correction methods remain undetermined [46,47]. The timing of urine collection also influences the results of urinary biomarkers. In the present study, subjects began exercising 30–60 min after pre-urine collection. Therefore, urinary biomarkers are also affected by urine accumulation in the bladder before exercise. Additionally, accumulating time of urine in the bladder following exercise also affects the results of organ damage markers and IRFs in urine because organ damage markers and IRFs in the circulation are excreted into urine constitutively. As described above, many factors influence organ damage markers and IRFs of spot urine.

### 4.3. Limitation

The limitation of this study is the validation of urinary biomarkers in exercise. In the present study, we did not measure the biomarkers in the circulation. Therefore, it is unclear to what degree urinary biomarkers reflect the change of circulating biomarkers. Another limitation is that, while the two time points utilized would have captured very acute responses, a longer temporal examination was not possible. Our study design was not enough to investigate organ damage and inflammatory responses in urine because many IRFs and organ damage markers change not only immediately, but also several hours after exercise [2,10]. Future studies are required over extended post-exercise durations to investigate these aspects further. While a key strength of this study is the broad range of species of biomarkers that were measured, our analysis did not extend to a proteomics approach. Therefore, other important biomarkers of relevance may not have been detected and an “omics” approach within future investigations is warranted.

### 4.4. The Potential Applications in Sports Field

Our findings can be applied to evaluating recovery after exercise and training. For example, an appropriate recovery period between training can be established based on not only subjective symptoms, such as fatigue, but also biochemical indicators. From the perspective to avoid overtraining, titin N-terminal fragments may be a useful biomarker because that is a muscle damage marker. Dehydration is one of the factors that exacerbate the exercise-induced inflammatory response [48,49], and severe systemic inflammation is one of the pathologies of heat stroke [50,51]. In the sports scene, loss of body weight is a major indicator of dehydration. In addition to this indicator, monitoring the inflammatory state using urinary IRFs is meaningful to avoid heat stroke (ideally, a cutoff value is determined).

## 5. Conclusions

A 3000-m time trial induced dramatic changes in various urinary biomarkers of organ damage and inflammation, but not biomarkers of oxidative stress and bone turnover. These results suggest organ damage markers and IRFs in urine have the potential to act as non-invasive indicators to evaluate organ functions in the exercise.

## Data Availability

The data presented in this study are available upon request from the corresponding author. The data are not publicly available.

## Supplementary Materials

The following are available online at https://www.mdpi.com/2076-3921/10/1/79/s1. Table S1: Changes of urinary organ damage markers after the 3000-m time trial.; Table S2: Changes of urinary inflammation-related factors after the 3000-m time trial.; Table S3: Changes of urinary oxidative stress markers after the 3000-m time trial.; Table S4: Changes of urinary metabolites after the 3000-m time trial.; Table S5: Changes of urinary bone resorption markers and minerals after the 3000-m time trial.
